# The Role of Skin and Orogenital Microbiota in Protective Immunity and Chronic Immune-Mediated Inflammatory Disease

**DOI:** 10.3389/fimmu.2017.01955

**Published:** 2018-01-10

**Authors:** Young Joon Park, Heung Kyu Lee

**Affiliations:** ^1^Graduate School of Medical Science and Engineering, Korea Advanced Institute of Science and Technology (KAIST), Daejeon, South Korea; ^2^KAIST Institute for Health Science and Technology, Korea Advanced Institute of Science and Technology (KAIST), Daejeon, South Korea

**Keywords:** immune response, microbiota, skin mucosa, orogenital mucosa, inflammation

## Abstract

The skin and orogenital mucosae, which constitute complex protective barriers against infection and injuries, are not only the first to come into contact with pathogens but are also colonized by a set of microorganisms that are essential to maintain a healthy physiological environment. Using 16S ribosomal RNA metagenomic sequencing, scientists recognized that the microorganism colonization has greater diversity and variability than previously assumed. These microorganisms, such as commensal bacteria, affect the host’s immune response against pathogens and modulate chronic inflammatory responses. Previously, a single pathogen was thought to cause a single disease, but current evidence suggests that dysbiosis of the tissue microbiota may underlie the disease status. Dysbiosis results in aberrant immune responses at the surface and furthermore, affects the systemic immune response. Hence, understanding the initial interaction between the barrier surface immune system and local microorganisms is important for understanding the overall systemic effects of the immune response. In this review, we describe current evidence for the basis of the interactions between pathogens, microbiota, and immune cells on surface barriers and offer explanations for how these interactions may lead to chronic inflammatory disorders.

## Introduction

The skin and mucosae constitute complex protective barriers against infection and injuries. Oral and genital mucosae differ from those of lung or gut as they are in constant contact with extrinsic stimulation, such as food, medication, and physical trauma. The skin and orogenital mucosae are similar with respect to function, as these barriers are not only the body’s first line of defense against pathogens but also hosts for a substantial number of commensals, including bacteria, fungi, and viruses. These organisms influence the immune response at the barrier sites and can lead to aberrant responses and chronic inflammation. Additionally, dysbiosis caused by specific bacteria species in the skin and orogenital mucosae is associated with tissue-specific chronic immune-mediated disease. The specific species responsible for this disease include *Staphylococcus aureus* on skin, *Porphyromonas gingivalis* on oral mucosa, and *Gardnerella vaginalis* on vaginal mucosa. The association between certain systemic diseases and changes in the microbiota of these barrier surfaces has just recently been reported, due to the fact that the microbiota in these areas is given less attention compared to the microorganisms residing in the gut. Understanding the interactions between the local commensals and the immune system at these barrier sites is important, however, as the initial interaction occurs at these sites and can lead to widespread disease. Thus, in this review, we describe the major immune mediators of interactions between the surface barrier tissue and local microorganisms with respect to the representative disease.

## Skin Microbiota

Human skin, which comprises the body’s largest organ, is home to many commensals. Across the 1.8 m^2^ of the skin surface, one million bacteria reside on each square centimeter, yielding a total of more than 10^10^ bacterial cells on the human body (1). While the gut supports 10^14^ bacterial cells per healthy individual, the skin contains the second highest number and diversity of bacterial cells with as many as 40 different species per individual (2, 3). The difference between skin microbiota and that of the large intestine stems largely from the direct contact of the skin with the surrounding environment. The combination of the multiple niches with various pH and temperatures created by these interactions with the environment and the diversity of the epidermal compositions and dermal appendages of the skin leads to the differences and diversity of the resident bacteria. The initial microbial skin colonization depends on the delivery mode. Vaginal delivery results in babies with microbiota similar to that of mother’s vagina, whereas babies born *via* cesarean section acquire microbiota related to skin. During puberty, the skin microbiota goes through a major transition with domination of lipophilic bacteria, such as *Propionibacterium* and *Corynebacterium*.

### The Skin Immune System

The immune system of the skin is composed of complex network of keratinocytes and immune cells, including the skin-specific antigen-presenting cells, the Langerhans cells. As the first line of defense against infection, the innate and adaptive immune systems are delicately controlled, and even a slight interference to the network can initiate an inflammatory response. The immune-microbial interaction is therefore quite important, despite the fact that the skin microbiota are not required for immune system organization (4). When in symbiosis, which is defined as a persistent balanced state between the skin and skin-resident microorganisms, a specific member of the microbiota acts to preserve barrier functions, but when the barrier is breeched by intrinsic or extrinsic factors, the very same member can initiate an immune response.

### Skin Microbiota and Innate Immunity

**Figure 1:**
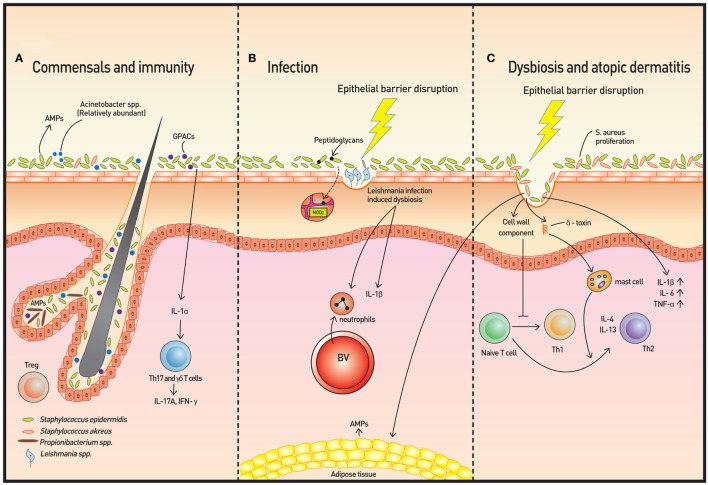
Skin microbiota and immunity. **(A)** The microbiome is more diverse in healthy skin. *Staphylococcus epidermidis, Acinetobacter* spp., and Gram-positive anaerobe cocci (GPAC) exhibit protective features against atopic disease. Keratinocytes and sebocytes release antimicrobial peptides (AMPs), and associations with skin commensals, such as *Propionibacterium* spp., have been demonstrated. Pattern recognition receptors (PRRs), such as nucleotide-binding oligomerization domain containing 2, recognize bacterial peptidoglycans to increase AMP production. Skin commensals also control the expression of interleukin (IL)-1, and increased IL-1 production is followed by IL-17A and subsequent interferon-γ production by dermal T helper 17 and γδ T cells. Regulatory T (Treg) cells reside primarily around fair follicles and interact with commensals within a specific time window to achieve immune tolerance. In addition to classical Foxp3^+^ Treg cells, Foxp3^−^ Treg cells also interact with the bacterium *Vitreoscilla filiformis* to induce Foxp3^−^ Treg cell differentiation. **(B)** The dysbiosis induced by Leishmaniasis infection is not only transmissible but also exacerbates skin inflammation *via* neutrophil recruitment and production of IL-1β. **(C)** In atopic dermatitis, *Staphylococcus aureus* proliferates and microbial diversity decreases concomitantly. Additionally, along with epithelial barrier disruption, proinflammatory cytokines are produced. Activation of T cells to Th2 cells occur *via* two mechanisms: degranulation of mast cells from δ-toxin and downregulation of IP-10 and other Th1 cell-recruiting chemokines.

Epidermal keratinocytes release antimicrobial peptides (AMPs), such as cathelicidins and β-defensins, which comprise the majority of these AMPs (Figure 1A). The AMPs, which are also produced by sebocytes, provide microbicidal activity against pathogens and may also trigger the inflammatory response. Some of these AMPs are actually controlled by the microbiota. For example, *Propionibacterium* species induce the expression of AMPs in human sebocytes (5).

In addition to microbiota regulation of AMPs, adipose tissue that interfaces with the skin may also contribute to the innate responses. In invasive *S. aureus* infection, local preadipocytes rapidly proliferate, leading to an expansion of the dermal fat layer, and simultaneously, cathelicidin is markedly increased in the adipose tissue and the infected skin (6). This response by the local adipose tissue provides both physical and immunologic antimicrobial defense.

Another response of the skin cells to bacterial pathogens is *via* pattern recognition receptors (PRRs). Nucleotide-binding oligomerization domain containing 2 (NOD2), an intracellular PRR, recognizes bacterial peptidoglycans of both Gram-positive and Gram-negative bacteria. Experimental manipulation of NOD2 led to bacterial dysbiosis with local changes in AMPs (7). Hence, although the specific mechanism has yet to be elucidated, NOD2-mediated immune surveillance appears to function in the immune response in association with the cutaneous microbiota.

Skin-resident microbes are also capable of regulating components of the complement system. Mice deficient in complement develop altered skin microbial communities (8). As inhibition of complement component C5a receptor in germ-free mice leads to decreased levels of AMPs and proinflammatory factors, the skin microbiome closely interacts with the complement system.

A recent study using a Leishmaniasis skin infection model demonstrated that human skin microbiota is altered following infection with *Leishmania* and that this dysbiosis is transmissible and capable of exacerbating skin inflammation *via* recruitment of neutrophils and production of interleukin (IL)-1β [Figure 1B (9)]. The skin microbiota also controls expression of IL-1α independently (4). The IL-1 cytokine is essential for the initiation and amplification of immune responses, and thus, the acute immune response is considered to be under the influence of host skin commensal interactions.

### Skin Microbiota and Adaptive Immunity

Skin microbiota is capable of promoting both the innate and the adaptive immune responses to limit pathogen invasion and maintain homeostasis. Mice without adaptive immunity fail to control their skin microbiota, allowing pathogenic microbial invasion to occur (10). The adaptive response, however, does not occur alone and is an extension of the innate response. For example, increased IL-1 production is followed by production of IL-17 and interferon-γ (IFN-γ) from dermal T cells. Reciprocally, T helper 17 (Th17) cells along with IL-17A-producing γδ T cells are reduced in mice lacking IL-1R1 (4). Introduction of *Staphylococcus epidermidis* to germ-free mice restores the production of IL-17A, indicating that *S. epidermidis* as part of the skin commensal microbiome potently induces Th17 cells as well as other T cells that express IL-17A. Other human skin commensals, such *as Corynebacterium pseudodiphtheriticum, Propionibacterium acnes, and Staphylococcus aureus*, also increase the number of skin IL-17A^+^ cells and IFN-γ^+^ cells but do not induce as prominent a response as the introduction of *S. epidermidis*. In addition, *S. epidermis* is the only commensal species that increased the frequency of CD8^+^ T cells in the skin. These CD8^+^ T cells are capable of producing either IFN-γ or IL-17A and homed only to the epidermis. These commensal-specific IL-17A-producing T cells successfully enhance barrier immunity and limit pathogen invasion (11). Skin-resident Th17 cells that are affected by the skin microbiota are also independent of those from the gut microbiota, suggesting that Th17 cells of the barrier sites are regulated in a “compartmentalized” manner by local commensals (4).

In the skin of both mice and humans, Foxp3^+^ regulatory T (Treg) cells are present in the dermis, especially surrounding the hair follicles, where skin-resident microorganisms also reside (12). Occupation of these hair follicles by commensals may be coupled with a regulatory response by Treg cells to limit abnormal inflammatory response against them (13). The time window during which this commensal-specific tolerance is achieved may be as early as the neonatal period. Precolonization of neonatal mice with *S. epidermidis* suppresses skin inflammation upon *S. epidermidis* challenge. A wave of Foxp3^+^ Treg cell infiltration in the skin occurs in the second week in neonate mice, and this infiltration is accompanied by higher levels of CTLA-4 and ICOS, which are both critical mediators of immune tolerance (14). The skin of germ-free mice, however, has a high frequency of Foxp3^+^ Treg cells compared with the skin of specific pathogen-free (SPF) mice (4). Thus, the underlying mechanism of Tregs in controlling host-microorganism dialog has yet to be fully elucidated. Foxp3^+^ Treg cells are known for their function in promoting microbial persistence (15), but Foxp3^−^ Treg cells may also be involved in establishing immune tolerance of certain microbiota. *Vitreoscilla filiformis*, a Gram-negative bacterium, induces dendritic cells to prime naïve T cells to type 1 Treg cells without Foxp3 expression. Furthermore, epicutaneous application of *V. filiformis* lysate induces IL-10^high^ T cells and inhibits T-cell proliferation in NC/Nga mice that exhibit atopic dermatitis (AD)-like inflammation (16). Nonetheless, as Treg cells are not the sole inducers of immune tolerance, much remains to be revealed about the basis of tolerance of constitutive commensals.

## Atopic Dermatitis

Atopic dermatitis is a prototype immune-mediated inflammatory skin disorder with prominent association with commensals. AD is considered to be an initiating stage of abnormal systemic Th2 response that progresses to allergic rhinitis and asthma. Some *Staphylococcus* spp., and in particular, *S. aureus*, have been found to be associated with AD and AD flares. Colonization of *S. aureus* on barrier-disrupted murine skin increases expression of IL-1β, IL-6, and TNF-α, demonstrating a pivotal role of *S. aureus* in promoting skin inflammation [Figure 1C (17)]. *S. aureus* colonization results in a Th2 immune response instead of a Th1 response despite the presence of its superantigen, which elicits a predominant Th1 cytokine profile, has been explained by two studies. First, δ-toxin released by *S. aureus* induces the degranulation of dermal mast cells and in turn, promotes the Th2 response (18). Second, components of the *S. aureus* cell wall downregulate IP-10 independent of IL-10; trigger activation of MAPK, p38, and ERK; and inhibit STAT1 signaling in monocytes, contributing to the abrogation of Th1 cell-recruiting chemokines (19). In contrast, *S. epidermidis* colonization provides innate immune signals to establish a functional threshold for adaptive immunity to facilitate pathogen control (20). As expected, overabundant colonization of *S. aureus* correlates with worsening AD in both mice and humans, but whether the loss of microbiome diversity leads to skin flares or skin flares result in reduced microbiotic diversity has not been illuminated (21). Dysbiosis with abundant *Staphylococcus* spp. and *Corynebacterium* spp., however, was observed in a genetically engineered murine model of AD. Also, administration of antibiotics to mice deficient in epidermal ADAM17 (Adam17^ΔSox9^) prevented eczematous lesion development, prevented an increase in skin-infiltrating T-cell numbers, and paradoxically, increased diversity of the skin microbiome. The study clearly showed that dysbiosis and overabundance of *S. aureus* contribute to AD-like lesions and are likely responsible for acute atopic flares in humans (22). The most recent reports also reveal a role for *S. aureus* in acute AD and new-onset pediatric AD, both of which show Th17 immune polarization. Epicutaneous *S. aureus* exposure induces skin inflammation by triggering IL-36R/MyD88 signaling and consequent IL-17 production from T cells (23, 24). The findings provide an explanation of increased IL-36α/γ transcripts and the increased number of Th17 cells in human AD skin (25, 26). On the other hand, skin commensals, including *S. epidermidis*, were found to inhibit *S. aureus via* production of AMPs that selectively kill *S. aureus*, and reintroduction of these commensals to human subjects decreased colonization by *S. aureus*, confirming the role of *S. epidermis* to prevent dysbiosis and initiation of inflammation (27). In addition to *S. epidermidis, Acinetobacter* species and Gram-positive anaerobe cocci (GPAC) may also play a protective role. *Acinetobacter* species were found to not only be more abundant in healthy subjects compared to atopic subjects but also to be associated with expression of anti-inflammatory molecules by peripheral blood mononuclear cells. *Acinetobacter* species induced strong Th1 and anti-inflammatory responses by immune cells and skin cells, suppressing allergic sensitization and lung inflammation in the skin (28). The abundance of GPAC was found to be low in human skin with a filaggrin deficiency, an important factor involved in AD pathogenesis. The cocci generated an inflammatory response distinct from that of *S. aureus in vitro* (29). These studies suggest that the loss of microbiome diversity is linked with a higher allergic response and subsequent inflammation.

## Oral Microbiota

**Figure 2:**
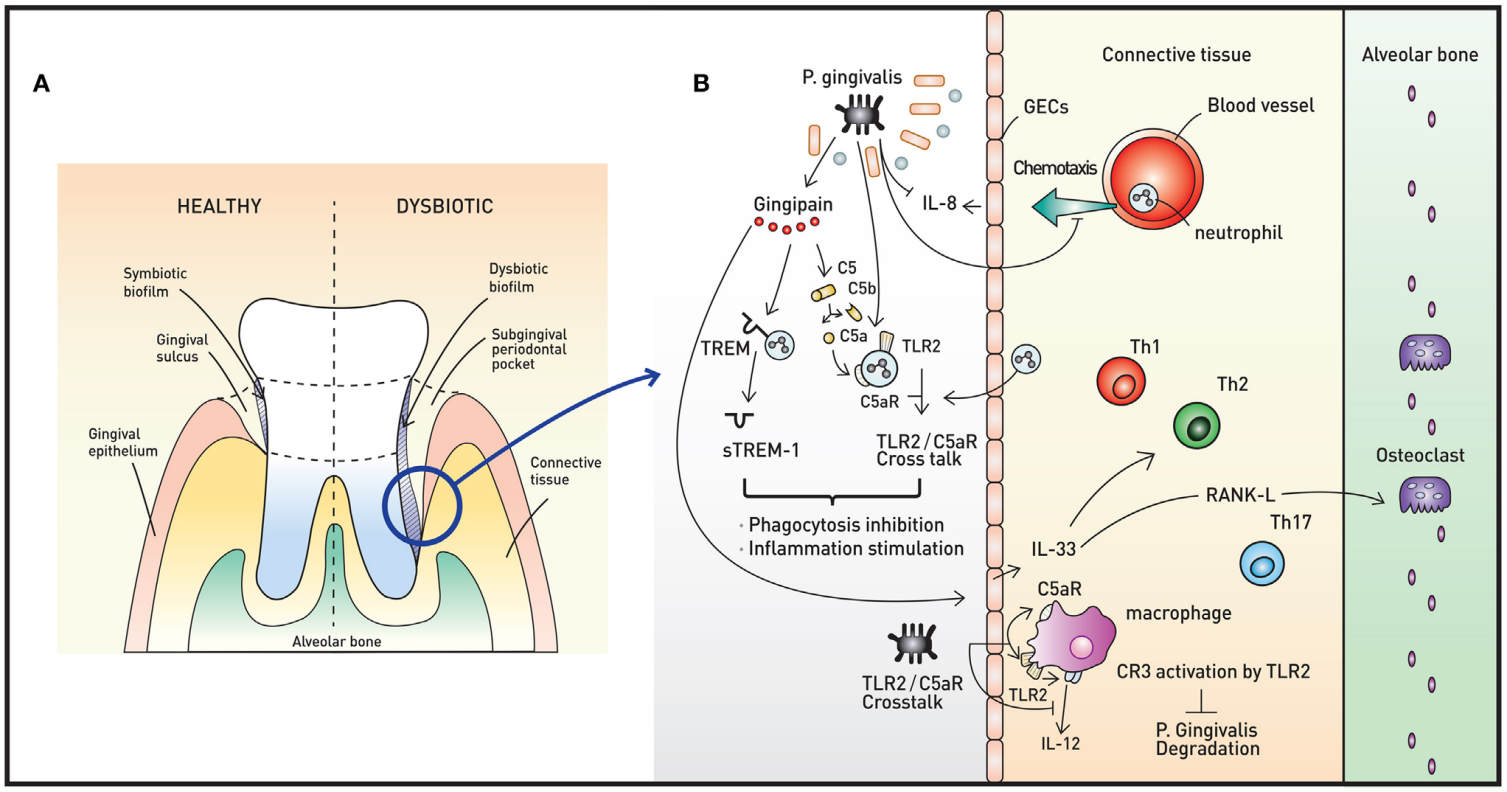
Oral microbiota and periodontal immunity. **(A)** The periodontium in healthy (left) and disease (right) states. When dysbiotic biofilm causes chronic inflammation of the gingiva, repeated swelling and inflammation results in deepening of the gingival sulcus, forming a subgingival periodontal pocket. The environment of the periodontal pocket intensifies the dysbiosis, and periodontal pathogens emerge. **(B)** A representative pathogen, *Porphyromonas gingivalis*, causes various immune responses that result in immune subversion and periodontitis. This organism inhibits secretion of IL-8 from gingival epithelial cells (GECs), interfering with the chemotaxis of neutrophils to the site of infection. *Via* the cysteine protease gingipain, *P. gingivalis* activates C5AR and toll-like receptor 2 (TLR2), triggering C5aR-TLR2 crosstalk. The crosstalk and cleavage of triggering receptor expressed on myeloid cells 1 (TREM-1) to soluble TREM-1 by gingipains result in simultaneous inhibition of phagocytosis and stimulation of inflammation. Gingipains also increase IL-33 production from GECs, downregulating antimicrobial peptides (AMPs) and increasing osteoclastogenecity. In macrophages, the C5aR-TLR2 crosstalk works to inhibit *P. gingivalis* degradation by suppressing production of nitric oxide and IL-12 without disruption of proinflammatory properties. The downregulation of IL-12 is also potentiated by binding of *P. gingivalis* to complement receptor 3.

The oral microbiota is also an important part of the human microbiota. A minimum of 700 different species are present in the human oral cavity, inhabiting diverse locations such as the tongue, hard tissues, and dentures (Figure 2A) (30). Similar to the skin, the microbiota of the oral cavity differs substantially based on the ecological niche (31). One unique characteristic of the oral microbiota is that due to saliva, which washes the mucosa and soft tissue surfaces, only a non-pathogenic monolayer exists on these surfaces. On the contrary, biofilm develops on hard tissue surfaces. Biofilm is an aggregate of extremely heterogeneous bacteria and contains sufficient nutrients to sustain the metabolic needs of the microbiota (32). Dental plaque, which is a typical form of biofilm, is known for its potential to induce inflammation. Under certain circumstances, for example, a change in pH, oxygen tension, or host immune status, the resident microbiota of the biofilm can transform to a pathogenic population (33). Pathogenic colonization with or without mucosal epithelial disruption impairs host immune responses and may result in disease, such as gingivitis or periodontitis.

The majority of bacteria in the oral cavity belong to five phyla: *Actinobacteria, Bacteroidetes, Firmicutes, Fusobacterium*, and *Proteobacteria*. As the microbial communities from different oral sites are distinct, the salivary microbiome is generally used as a representative microbiome (34). In a Japanese study on the salivary microbiome of 2,343 adults aged 40 years or older, the population could be divided in to two groups based on the composition of the microbiome. The group with more abundant *Prevotella histicola, Prevotella melaninogenica, Veillonella parvula, Veillonella atypica, Streptococcus salivarius*, and *Streptococcus parasanguinis* was associated with poorer oral hygiene and poorer general conditions, suggesting that the oral microbiota reflects both the local and the systemic immune status (35). Despite the characterization of the overall composition of the oral microbiome (36), studies on the immunologic role of each organism in the oral microbiome, notably bacteria without virulence factors, are remarkably scarce. The diversity of the oral microbiome based on spatial niches and constant temporal change due to its nature as an open system exposed to exogenous microbes may underlie the rarity of such studies, although a few studies have been completed. One example of such a study showed that bacteriocin produced by *S. salivarius* inhibits the growth of Gram-negative species associated with periodontitis and halitosis *in vitro* (37), in agreement with evidence that *S. salivarius* as a probiotic is beneficial to halitosis *in vivo* (38, 39).

## Periodontal Microbiome, Protective Immunity, and Periodontitis

The gingival sulcus and periodontal pocket are the most-studied niches of microbial colonization in the oral mucosa. The crevice between the hard surface of the teeth and the gingiva harbors microbial communities that interact with the mucosal epithelial cells. Phylum *Proteobacteria*, particularly the *gammaproteobacteriae* of genus *Acinetobacter, Haemophilus*, and *Moraxella*, were most prevalent in healthy gingival sulci less than 4 mm deep. Periodontal pockets are formed when the attachment between the gingivae and teeth is lost. The space then can become colonized with anaerobic bacteria. The microbiota highly associated with pockets greater than 4 mm in depth include *Spirochetes* genus *Treponema*; *Synergistetes* genus *Sinergistes*; *Bacteroidetes*, such as genera *Porphyromonas, Prevotella*, and *Tannerell*a; and *Fusobacteria* genera *Fusobacterium* and *Leptotrichia* (40, 41). Development of culture-independent techniques has expanded the range of associated species that were previously uncultivable or underappreciated. These species include *Filifactor alocis, Peptostreptococcus stomatis, Prevotella denticola, Porpyromonas endodontalis, Anaeroglobus geminatus*, and *Eubacterium saphenum* (40, 42).

Toll-like receptors (TLRs) and other PRRs act as immunologic sensors of the biofilm. The epithelial cells activated by microbe-associated molecular patterns (MAMPs) of the biofilm increase its proliferative rate, expression of adhesion molecules, IL-1β production, and production of AMPs, such as calprotectin (S100A8/A9) and defensins (27). Calprotectin from gingival epithelial cells (GECs) functions both extracellularly and intracellularly *via* incorporation into neutrophil extracellular traps and aiding innate intracellular immunity, respectively, providing resistance against pathogenic bacteria (31, 43).

In periodontal disease (also termed periodontitis), a chronic inflammatory disease that results in the destruction of bony apparatus, the components of the pattern recognition system, for example TLR2, initiate the sustained inflammation and ultimately induce the induction of bone loss in mice (44). Recently, the NOD1 cytoplasmic PRR was found to be responsible for the majority of periodontal bone destruction. Interestingly, monocolonization of germ-free animals with a single specific bacterium NI1060, which exhibits a greater than 60% homology with the coding sequences of *Aggregatibacter actinomycetemcomitans*, was sufficient to promote periodontal bone destruction in a NOD1-dependent manner (45). These findings demonstrate that a single bacterial component from a normal commensal microbiome can cause destructive disease through activation of a PRR in oral mucosa. Calprotectin is also abnormally activated in periodontitis and has been implicated in the pathogenesis of this disease (46).

T helper 17 cells have been implicated in mediating protective immunity as well as pathogenic inflammatory response at multiple barrier sites, including the oral cavity, skin, and gut. Gingival Th17 cells develop independently of commensal microbe colonization. As the frequencies of gingival Th17 cells are unchanged in germ-free mice compared to SPF mice, the oral microbiome does not appear to be a primary driver of gingival Th17 development, and this finding is in contrast to the situation in the skin and gastrointestinal tract. IL-6 expression in response to mechanical damage of epithelial cells instead is the major promoting factor for accumulation of Th17 cells and subsequent elevation of epithelial antimicrobial peptides and neutrophil chemo-attractants in the oral cavity (47). These data should be carefully interpreted, because it is evident that dysbiotic microbial communities aggravate periodontitis possibly in relation to accumulated Th17 cells (48–50). Mechanical damage may be critical for Th17 cell proliferation but also serves as an amplifier of the oral inflammatory response.

## *Porphryomonas* *gingivalis*

*Porphyromonas gingivalis*, a member of the phylum *Bacteroidetes*, is a keystone oral pathogen that evades the immune system and alters local host responses. While this Gram-negative anaerobic rod-shaped bacterium mainly resides in biofilms and behave as a commensal, it also invades host cells, such as GECs in certain situations. This bacterium occupies its own replicative niche within autophagosomes (51). This microorganism also underlies the “dysbiosis hypothesis,” which surmises that *P. gingivalis*, even at low colonization levels, triggers changes in the amount and composition of the oral commensal microbiota, leading to inflammation and ultimately periodontal bone loss. Interestingly, the commensal microbiota and complement are both required for this *P. gingivalis*-induced bone loss, as *P. gingivalis*, although necessary, is not sufficient to induce inflammation (52). Thus, *P. gingivalis* exerts its pathogenic effects *via* its ability to induce dysbiotic microbial communities (Figure 2B). Manipulation of the host immune system (called immune subversion) by *P. gingivalis*, therefore, is essential and is therefore discussed in detail in this review.

### *P. gingivalis* and Innate Immunity

As described above, PRRs, such as TLR2 and NOD1, are mediators of the innate immune response. *P. gingivalis* coactivates TLR2 and C5a receptor (C5aR) in neutrophils, resulting in crosstalk that leads to ubiquitination and proteasomal degradation of MyD88 and inhibits the host-protective antimicrobial response. Moreover, this C5aR-TLR2 crosstalk activates PI3K, which prevents phagocytosis through inhibition of RhoA activation and actin polymerization and stimulates an inflammatory response (53). Upon initiation of inflammation, GECs secrete the chemoattractant IL-8 to recruit neutrophils. *P. gingivalis* inhibits the secretion of IL-8 from GECs, lowering the number of neutrophils recruited to the site of inflammation. Another way that *P. gingivalis* inhibits the polymorphonuclear leukocyte function is through the binding of whole cells and lipopolysaccharides of the bacteria to adhesion molecules, interrupting leukocyte diapedesis. Other mechanisms to manipulate neutrophil activity include bacterial binding to fMLF receptor and PPAD-citrullinated C5a, resulting in inhibition of chemotaxis, and regulation of triggering receptor expressed on myeloid cells 1 (TREM-1) *via* both gingipain-dependent and -independent mechanisms, rendering the inflammatory response appropriate for the bacteria (54).

Gingipains are cysteine proteases that act as critical enzymes in periodontitis pathogenesis. In addition to cleavage of TREM-1 by Arg-gingipain, gingipain is responsible for proteasomal degradation of *P. gingivalis* and increased inflammation, as this enzyme cleaves C5 to C5a, leading to My88D degradation and PI3K activation (53). Gingipain is also associated with increased IL-33 production by GECs *via* PAR-2-p38/NF-κB signaling (55). IL-33 is an alarmin released in response to tissue damage, and increased IL-33 downregulates AMPs, such as LL-37 (56). Moreover, IL-33 induces receptor activator of NF-κB ligand, a crucial osteoclastogenic factor (57).

In macrophages, C5aR-TLR2 crosstalk by *P. gingivalis* suppresses production of nitric oxide and IL-12 simultaneously, inhibiting *P. gingivalis* killing but preserving the ability of these cells to elicit an inflammatory response (58, 59). *P. gingivalis* binds to complement receptor 3 (CR3) on macrophages to downregulate IL-12 selectively. This binding further allows *P. gingivalis* to persist intracellularly, as CR3 is not coupled to microbicidal mechanisms directly (60).

### *P. gingivalis* and Adaptive Immunity

Macrophages engage microbes not only to eliminate them but also to work as antigen-presenting cells. Currently, the M1 subpopulation of macrophages is considered to be responsible for inflammatory function, and *P. gingivalis* clearly triggers M1-type-associated inflammatory pathways (61, 62). A recent study, however, indicated a novel M1 macrophage appears in response to *P. gingivalis* with the addition of IFN-γ, which significantly upregulated proinflammatory cytokines, such as IL-1β, IL-6, and TNF-α, and lowered production of chemokines related to T-cell recruitment (IL-12, IL-23, and CXCL10). These effects are in stark contrast to those induced by *A. actinomycetemcomitans*, another representative oral pathogen. Thus, *P. gingivalis* may have a unique capacity to alter the programmed course of the hyperinflammatory and T-cell immunomodulatory M1 macrophages (63).

*Porphyromonas gingivalis* appeared to drive dendritic cells, however, toward a Th2 antigen-presenting cell phenotype, at least *in vitro* (64). A Th2 cytokine-mediated inflammatory response was observed in association with *P. gingivalis*, and IL-33 was again identified recently using *P. gingivalis* gingipain-null mutant KDP cells. Of the various virulence factors, gingipain was entirely responsible for the IL-33 increase that was observed in human GECs (55). As evaluation of the protein levels in cells activated by *P. gingivalis* revealed no obvious Th1- or Th2-skewed profiles (65) and IL-1β and IL-6 were produced by CD4^+^ T cells (66), the specific response of Th cells induced by this bacterium remains to be fully delineated. Notably, *P. gingivalis* was posited to stimulate myeloid antigen-presenting cells to drive Th17 polarization, and inactive gingipains selectively generated a Th17 phenotype in an IL-6-dependent manner. Inhibition of IL-6 signaling in dendritic cells led to a significant depletion of the Th17 population without similar effects on other T-cell subsets. These studies unveiled the possibility that Th17 cells are involved in the pathogenesis of periodontitis and confirmed that IL-6 signaling is an attractive target for treatment of this disease (49).

Despite the paucity of *in vivo* evidence of *P. gingivalis* manipulation of the adaptive immune response, this organism should still be considered a keystone species and successful pathogen with the ability to modulate adaptive immunity, as *P. gingivalis* disables the overall host response while simultaneously enhancing the inflammatory response and the pathogenicity of a polymicrobial community.

## Vaginal Microbiota

The human vaginal mucosa is home to abundant microflora. The environment is exposed to unique foreign substances, such as spermatozoa and sexually transmitted pathogens. The environment is also very versatile, constantly changing due to menstrual cycle changes of the female body. In addition, the composition of the microbial community is largely influenced by events such as pregnancy and menopause. The commensals of the vagina change throughout a woman’s life and function as a main component of the vaginal mucosal defense against pathogens.

**Figure 3:**
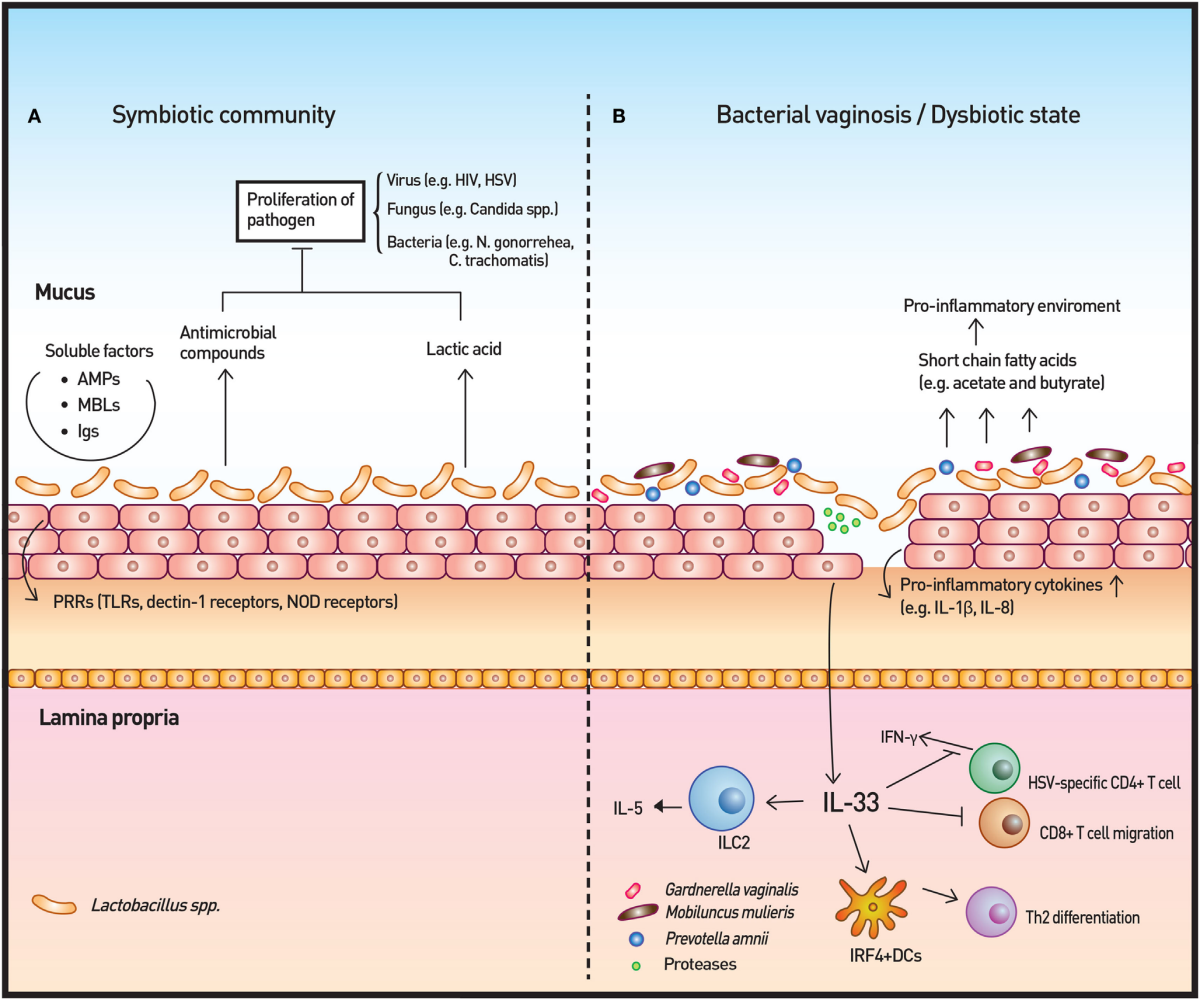
Vaginal microbiota and immunity. **(A)** The vaginal microbiota in a healthy individual is dominated by *Lactobacillus* spp. The *Lactobacillus* spp. produce lactic acid as well as antimicrobial compounds to control the growth of microbes. Other soluble factors, such as antimicrobial peptides (AMPs), mannose-binding lectins (MBLs), and immunoglobulins (Igs), contribute to the homeostatic immunity of the vaginal surface. In addition, the surveillance of commensals and pathogenic microbes is achieved by pattern recognition receptors (PRRs). **(B)** In cases of disrupted vaginal microbiota, such as bacterial vaginosis, community state type IV type microorganisms dominate to initiate an inflammatory response. Short-chain fatty acids produced by these microorganisms are likely to induce the production of proinflammatory cytokines. IL-33 has recently been identified as the key cytokine in association with antiviral immunity modulation by the vaginal microbiome. IL-33 is also responsible for the Th2-type immune response elicited by proteases that are secreted by pathogenic microbes.

Out of the ~200 bacterial species that reside in the vagina, *Lactobacillus* species predominantly colonize the vaginal tract (Figure 3A). Currently, more than 120 *Lactobacillus* species have been described and comprise more than 70% of resident bacteria in women. *Lactobacilli* are believed to contribute to the immunity in a healthy vagina. *Lactobacilli* produce significant amounts of lactic acid, ensuring the environment maintains a relatively low pH. Furthermore, these bacteria produce H_2_O_2_, which is presumed to contribute to protection of the vagina from pathogens, as H_2_O_2_-producing vaginal *Lactobacillus* spp. are more protective against bacterial vaginosis (BV) than those that do not produce H_2_O_2_ (67, 68).

The vaginal mucosa of a single woman is usually dominated by one or two species of *Lactobacillus*, and attempts to classify women by lactobacill*i* and other microbiota species have shown that ethnicity affects the microbiome. Caucasian women are dominated by *L. iners*, black and Hispanic women are dominated by *L. genseni*, and Asian women are dominated by *L. crispatus*. Similarly, reproductive-aged women have been classified to five community state types (CSTs). These types include women who harbored *L. crispatus* preferentially (CST I), women who were dominated by *L. gasseri* (CST II), and women who were dominated by *L. iners* (CST III). Furthermore, CST IV was initially defined as a complex mixture of bacteria, including *Prevotella*, without any Lactobacillus species dominance. Additionally, the presence of *L. crispatus* and/or *L. iners* without dominance was assigned as CST IV-A, and no or minimal lactobacilli was assigned as CST IV-B. The clinical significance of these groupings has yet to be fully explored, but once established, the vaginal microbiota is stable, despite frequent interruptions, such as sexual behavior (69, 70).

The vaginal microbiota is also influenced by gene polymorphisms, and the innate immune response to the microbiota in the female genital tract has been found to be associated with these genetic variants. Single-nucleotide polymorphisms that disrupt immune recognition are associated with increased susceptibility to disruption of vaginal microbiota and vaginal infections (71, 72). Especially, polymorphisms in IL-4, IL-10, TLR4, and TNF-α genes have been shown to induce aberrant responses to BV-associated bacteria and preterm delivery (72, 73). Of note, periodontal disease and BV are both influenced by gene polymorphisms and are both associated with preterm birth (74).

### Vaginal Microbiota and Protective Immunity

Pattern recognition receptors, including TLRs, dectin-1 receptors, and NOD receptors, work as inspectors for MAMP on both commensal and pathogenic microbes (75, 76). Upon binding, PRRs initiate cytokine/chemokine signaling cascades for host defense. The cytokine/chemokines, including IL-1β, IL-6, and IL-8, recruit and activate various immune cells, such as macrophages, natural killer cells, and T cells. *In vitro* colonization of vaginal epithelial cell multilayers by *Lactobacilli* demonstrated that these bacteria do not elicit an inflammatory response in contrast to *S. aureus*. Rather, isolates of *Lactobaclli*, especially *L. jensenii*, tempered the induction of cytokines following TLR2/6 and 3 agonist treatment of the epithelial cells (77). Within the CST IV classification, *Prevotella amnii, Mobiluncus mulieris, Sneathia amnii*, and *Sneathia sanguinegens* were found to induce higher levels of inflammatory cytokines and chemokines, such as IL-1α/β and IL-8, relative to microbiota dominated by *L. crispatus* or *L. iners*. The study also showed significant increases of IL-1α/β and TNF-α during transition of communities from CST I to CST IV longitudinally, supporting the notion that the innate immune response is affected by vaginal bacterial community states (78).

Furthermore, although the number of vaginal antigen-presenting cells (VAPCs) does not vary with vaginal microbial populations, the CST IV VAPCs were more activated and mature and induced a marked response to LPS as well to IFN-γ and IL-1β. The study suggests that VAPCs are stimulated by contact with the resident flora to upregulate inflammatory cytokine/chemokine expression, resulting in a significant increase in vaginal CCR5^+^ CD4^+^ T cells (78). The increase of the accessible CD4 T-cell is important, as the vaginal mucosa is a restricted space for lymphocyte migration (79).

Soluble factors such as mannose-binding lectin (MBL), vaginal AMPs, and immunoglobulins (Igs) also contribute to vaginal defense (80). MBL binding to mannose, N-acetylglucosamine and fucose carbohydrate moieties present on microbial cell surfaces leads to cell lysis or recognition by immune cells (81). Women with MBL deficiency due to genetic polymorphism are more susceptible to recurrent vulvovaginal candidiasis (82). Defensins are a class of AMPs that act against various pathogens, including bacteria and viruses. The concentration of defensin three is associated with dysbiosis and BV during pregnancy (83). Human β-defensin-2 expression was associated with colonization by *L. iners, Atopovium vaginae*, and *Prevotella bivia* in *ex vivo* organotypic models of the vaginal epithelium and with *L. jensenii* but not *G. vaginalis in vitro* (71, 84). Natural Igs, such as IgG, present in vaginal mucus prevent viral infections, such as HSV, by forming multiple low-affinity bonds between the virus and mucus gel. A sufficient number of low-affinity bonds ensures that viruses are effectively trapped in mucus, thereby reducing the flux of infectious virions (85).

*Lactobacilli* dominance of vaginal microbiota has been shown to protect the host from other opportunistic microbial infections, including bacteria (e.g., *Neisseria gonorrhoeae* and *Chlamydia trachomatis*), viruses (e.g., HIV and HPV), and fungi [e.g., *Candida albicans* (86–90)]. The lactic acid and antimicrobial compounds produced by *Lactobacilli* are hypothesized to underlie such protection, and many studies are now focused on discovering the specific mechanisms (90, 91). Notably, of the *Lactobacillus species, L. crispatus* is most prominently involved in inhibition of the growth of other microbes and in suppression of cytokine production, whereas *L. iners*, despite its affiliation with *Lactobacilli*, shows conflicting results.

## Bacterial Vaginosis

A disrupted vaginal microbiota may be related to various diseases but is directly connected with two pathogenic states, BV and aerobic vaginitis (AV) (Figure 3B). Both of these disease entities originate from the CST IV category. AV is mainly differentiated from BV by the presence of prominent inflammatory response associated with aerobes, such as group B *Streptococcus, S. aureus, E. coli*, and *Enterococcus* (80, 92). Despite prominent clinical symptoms and a higher risk of preterm labor and preterm birth than BV (93), the identity of AV as dysbiosis-induced inflammation is still controversial, as some believe that AV is primarily an immunologic disorder with secondary dysbiosis or a dermatological disease (94). Thus, we will focus our discussion on BV in this review.

Bacterial vaginosis is defined as a replacement of lactobacilli with characteristic groups of bacteria and subsequent change in vaginal fluid. Clinically, Amsel et al. proposed the first diagnostic criteria for BV: gray-white milky homogeneous discharge, vaginal fluid pH > 4.5, clue cells upon microscopy, and release of fishy odor on 10% potassium hydroxide solution (95). An alternative for diagnosis is to use a vaginal smear for Gram-staining of *Lactobaccillus, Gardnerella*, and other Gram-variable rods to yield a Nugent criteria (96). BV may or may not elicit overt inflammatory responses, and investigation of inflammatory cytokines in BV has led to inconsistent results (97).

Bacterial vaginosis has long been known to be associated with *G. vaginalis*; however, following the development of non-culture-based methods, it became evident that *G. vaginalis* is often present in the absence of BV, suggesting that colonization by this bacterium is not a precondition of BV. Furthermore, BV is not caused by transferring *G. vaginalis* to a healthy woman, but transfer of discharge from a woman with BV is enough to elicit BV. Hence, it is not a single type of bacteria, even at high numbers, that is important but a critical mixture of bacteria with potential pathogenic properties and a lack of favorable lactobacilli that is essential for the development of BV (98, 99).

### BV-Altered Immune Response

Short-chain fatty acids (SCFAs) produced by commensal microbiota play an anti-inflammatory role in the gut (100); however, high concentrations (20 mM) of some SCFAs (acetate and butyrate) that are prevalent in BV induce PBMC production of the proinflammatory cytokines IL-8, TNF-α, and IL-1β at neutral pH, which resembles the pH of vagina. Lower levels of these SCFAs also significantly enhance TLR2 ligand- and TLR7 ligand-induced production of IL-8 and TNF-α in a time- and dose-dependent manner (101). The discordance between the effect of SCFAs in the gut and the effect of SCFAs in the vagina may be ascribed to inherent differences in these organs, including cell-type, SCFA concentration, and pH (101, 102). *In vitro* studies using vaginal epithelial cells and BV-associated bacteria have shown a similar proinflammatory response to that elicited by BV-associated SCFAs (71, 77, 103). In addition, a number of studies have reported higher levels of proinflammatory cytokines, such as IL-1β and IL-8, in women with BV than in controls with normal Nugent scores (104, 105). Taken together, these studies indicate that these metabolites may contribute to BV and the sub-clinical inflammation present in women with BV.

With respect to altered T-cell responses, CD4^+^, CD4^+^ CCR5^+^, and CD4^+^ CD69^+^ T cells were found to be decreased in the cervixes of Kenyan sex workers following BV treatment (106). Gamma delta T cells in the endocervix were also decreased in women with BV (107). On the contrary, qPCR-confirmed presence of both *L. crispatus* and *L. jensenii* was associated with lower numbers of cervical CD3^+^ HLADR^+^ and CD3^+^ CD4^+^ CCR5^+^ cells in healthy Belgian women (108). These results suggest a connection between BV and T cells, although the specific details of this relationship warrant further research.

Another important consequence of BV-altered immunity is the increased risk of viral infection, including sexually transmitted viruses, such as HIV and HSV. Commensal *Lactobacilli* inhibit HIV-1 replication in human tissue *ex vivo* by medium acidification, lactic acid production, and direct viricidal effects (89). The proinflammatory cytokines and chemokines that are increased by BV-associated bacteria *in vitro* and are associated with BV *in vivo* enhance the risk of HIV transmission by directly stimulating HIV replication in latent viral reservoirs and by facilitating the trafficking and activation of CD4^+^ host cells, which are normally sparse in the cervicovaginal mucosa (88). A recent study highlighted the importance of IL-33 in antiviral immunity of vaginal mucosa. The study demonstrated that the innate immune responses, including type I IFN and proinflammatory cytokine production at infection sites as well as induction of virus-specific CD4 and CD8 T-cell responses in draining lymph nodes, were not impaired when vaginal dysbiosis was induced using oral antibiotics. IL-33 alone was able to suppress local antiviral immunity by blocking the migration of effector T cells to the vaginal tissue, thereby inhibiting IFN-γ production (109). The study solidified the relationship between dysbiosis and HSV infection and revealed the mechanisms through which dysbiosis modulates antiviral immunity. The researchers further investigated the T-cell response from bacteria- or protozoan-secreted proteases. Challenge with the prototype protease papain on vaginal mucosa induced Th2 immunity that was dependent on IL-33. Furthermore, dendritic cells that express interferon regulatory factor 4 were responsible for the induction of Th2 differentiation (110).

## Concluding Remarks

Skin, oral, and genital mucosa function similarly as the initial barriers of host defense from pathogens; however, the interactions of these mucosae with microbes and the microbial rendering of these environments are unique, which can also be described as “compartmentalized” (4). Still, researchers should not overlook the possibility of connections between the microbiota of different tissues. Recently, ectopic colonization of oral bacteria in the intestine was found to induce gut inflammation (111). Diseases, such as Behçet’s disease, which affects orogenitalia, gut, and skin, exhibit distinct salivary microbiota as well as gut microbiota (100, 112). Thus, despite the absence of continuity, it remains feasible that the microbiota of one tissue affects another tissue.

The interaction between the major pathogenic microbes and subsequent chronic inflammatory immune response sheds light on the diverse mechanisms of the host response against dysbiosis and chronic systemic disorders. In addition, these microbes with pathogenic potential can become more than commensals, and thus, illuminating the key metabolic alterations responsible for their expansion will increase our understanding of tissue-specific pathology.

*In vivo* studies describing the host adaptive immunity and its association with compartmentalized microbiota outside of the gut remain scarce, likely due to the difficulty of such research. Skin and orogenital mucosa are profound reservoirs of T cells, especially tissue-resident memory T (TRM) cells (12, 113–115), which are cells recently identified as lymphocytes that are distinct from recirculating central and effector memory T cells (116). Thus, studies that illustrate the relationship between the microbiota and TRM cells are very likely to increase our understanding of the continuous interaction between our body and microbes. Moreover, novel strategies targeting the host manipulation of microbes with immune cells can also be developed *via* information we learn from further studies on microbiota and the human immune system.

## Author Contributions

YJ performed literature research and wrote the review. HK conceived the idea for the review, provided insightful discussion when necessary and edited the review.

## Conflict of Interest Statement

The authors declare that the research was conducted in the absence of any commercial or financial relationships that could be construed as a potential conflict of interest.

## References

[1] Grice EA Kong HH Renaud G Young AC NISC Comparative Sequencing Program Bouffard GG A diversity profile of the human skin microbiota. Genome Res (2008) 18(7):1043–50. 10.1101/gr.075549.107 18502944 PMC2493393

[2] Belkaid Y Segre JA. Dialogue between skin microbiota and immunity. Science (2014) 346(6212):954–9. 10.1126/science.1260144 25414304

[3] Honda K Littman DR. The microbiome in infectious disease and inflammation. Annu Rev Immunol (2012) 30:759–95. 10.1146/annurev-immunol-020711-074937 22224764 PMC4426968

[4] Naik S Bouladoux N Wilhelm C Molloy MJ Salcedo R Kastenmuller W Compartmentalized control of skin immunity by resident commensals. Science (2012) 337(6098):1115–9. 10.1126/science.1225152 22837383 PMC3513834

[5] Nagy I Pivarcsi A Kis K Koreck A Bodai L McDowell A Propionibacterium acnes and lipopolysaccharide induce the expression of antimicrobial peptides and proinflammatory cytokines/chemokines in human sebocytes. Microbes Infect (2006) 8(8):2195–205. 10.1016/j.micinf.2006.04.001 16797202

[6] Zhang LJ Guerrero-Juarez CF Hata T Bapat SP Ramos R Plikus MV Innate immunity. Dermal adipocytes protect against invasive *Staphylococcus aureus* skin infection. Science (2015) 347(6217):67–71. 10.1126/science.1260972 25554785 PMC4318537

[7] Williams H Crompton RA Thomason HA Campbell L Singh G McBain AJ Cutaneous NOD2 expression regulates the skin microbiome and wound healing in a murine model. J Invest Dermatol (2017) 137(11):2427–36. 10.1016/j.jid.2017.05.029 28647345 PMC5646944

[8] Chehoud C Rafail S Tyldsley AS Seykora JT Lambris JD Grice EA. Complement modulates the cutaneous microbiome and inflammatory milieu. Proc Natl Acad Sci U S A (2013) 110(37):15061–6. 10.1073/pnas.1307855110 23980152 PMC3773768

[9] Gimblet C Meisel JS Loesche MA Cole SD Horwinski J Novais FO Cutaneous leishmaniasis induces a transmissible dysbiotic skin microbiota that promotes skin inflammation. Cell Host Microbe (2017) 22(1):13–24.e4. 10.1016/j.chom.2017.06.006 28669672 PMC5555377

[10] Shen W Li W Hixon JA Bouladoux N Belkaid Y Dzutzev A Adaptive immunity to murine skin commensals. Proc Natl Acad Sci U S A (2014) 111(29):E2977–86. 10.1073/pnas.1401820111 25002505 PMC4115524

[11] Naik S Bouladoux N Linehan JL Han SJ Harrison OJ Wilhelm C Commensal-dendritic-cell interaction specifies a unique protective skin immune signature. Nature (2015) 520(7545):104–8. 10.1038/nature14052 25539086 PMC4667810

[12] Sanchez Rodriguez R Pauli ML Neuhaus IM Yu SS Arron ST Harris HW Memory regulatory T cells reside in human skin. J Clin Invest (2014) 124(3):1027–36. 10.1172/JCI72932 24509084 PMC3934172

[13] Belkaid Y Tamoutounour S. The influence of skin microorganisms on cutaneous immunity. Nat Rev Immunol (2016) 16(6):353–66. 10.1038/nri.2016.48 27231051

[14] Scharschmidt TC Vasquez KS Truong HA Gearty SV Pauli ML Nosbaum A A wave of regulatory T cells into neonatal skin mediates tolerance to commensal microbes. Immunity (2015) 43(5):1011–21. 10.1016/j.immuni.2015.10.016 26588783 PMC4654993

[15] Belkaid Y Piccirillo CA Mendez S Shevach EM Sacks DL. CD4+CD25+ regulatory T cells control *Leishmania major* persistence and immunity. Nature (2002) 420(6915):502–7. 10.1038/nature01152 12466842

[16] Volz T Skabytska Y Guenova E Chen KM Frick JS Kirschning CJ Nonpathogenic bacteria alleviating atopic dermatitis inflammation induce IL-10-producing dendritic cells and regulatory Tr1 cells. J Invest Dermatol (2014) 134(1):96–104. 10.1038/jid.2013.291 23812300

[17] Wanke I Skabytska Y Kraft B Peschel A Biedermann T Schittek B. *Staphylococcus aureus* skin colonization is promoted by barrier disruption and leads to local inflammation. Exp Dermatol (2013) 22(2):153–5. 10.1111/exd.12083 23362876

[18] Nakamura Y Oscherwitz J Cease KB Chan SM Munoz-Planillo R Hasegawa M *Staphylococcus* delta-toxin induces allergic skin disease by activating mast cells. Nature (2013) 503(7476):397–401. 10.1038/nature12655 24172897 PMC4090780

[19] Li Z Levast B Madrenas J. *Staphylococcus aureus* downregulates IP-10 production and prevents Th1 cell recruitment. J Immunol (2017) 198(5):1865–74. 10.4049/jimmunol.1601336 28122962

[20] Biedermann T Skabytska Y Kaesler S Volz T. Regulation of T cell immunity in atopic dermatitis by microbes: the Yin and Yang of cutaneous inflammation. Front Immunol (2015) 6:353. 10.3389/fimmu.2015.00353 26217343 PMC4500098

[21] Kong HH Oh J Deming C Conlan S Grice EA Beatson MA Temporal shifts in the skin microbiome associated with disease flares and treatment in children with atopic dermatitis. Genome Res (2012) 22(5):850–9. 10.1101/gr.131029.111 22310478 PMC3337431

[22] Kobayashi T Glatz M Horiuchi K Kawasaki H Akiyama H Kaplan DH Dysbiosis and *Staphylococcus aureus* colonization drives inflammation in atopic dermatitis. Immunity (2015) 42(4):756–66. 10.1016/j.immuni.2015.03.014 25902485 PMC4407815

[23] Liu H Archer NK Dillen CA Wang Y Ashbaugh AG Ortines RV *Staphylococcus aureus* epicutaneous exposure drives skin inflammation via IL-36-mediated T cell responses. Cell Host Microbe (2017) 22(5):653–66.e5. 10.1016/j.chom.2017.10.006 29120743 PMC5774218

[24] Nakagawa S Matsumoto M Katayama Y Oguma R Wakabayashi S Nygaard T *Staphylococcus aureus* virulent PSMalpha peptides induce keratinocyte alarmin release to orchestrate IL-17-dependent skin inflammation. Cell Host Microbe (2017) 22(5):667–77.e5. 10.1016/j.chom.2017.10.008 29120744 PMC5728420

[25] Suarez-Farinas M Ungar B Correa da Rosa J Ewald DA Rozenblit M Gonzalez J RNA sequencing atopic dermatitis transcriptome profiling provides insights into novel disease mechanisms with potential therapeutic implications. J Allergy Clin Immunol (2015) 135(5):1218–27. 10.1016/j.jaci.2015.03.003 25840722

[26] Koga C Kabashima K Shiraishi N Kobayashi M Tokura Y. Possible pathogenic role of Th17 cells for atopic dermatitis. J Invest Dermatol (2008) 128(11):2625–30. 10.1038/jid.2008.111 18432274

[27] Nakatsuji T Chen TH Narala S Chun KA Two AM Yun T Antimicrobials from human skin commensal bacteria protect against *Staphylococcus aureus* and are deficient in atopic dermatitis. Sci Transl Med (2017) 9(378). 10.1126/scitranslmed.aah4680 PMC5600545 28228596

[28] Fyhrquist N Ruokolainen L Suomalainen A Lehtimaki S Veckman V Vendelin J *Acinetobacter* species in the skin microbiota protect against allergic sensitization and inflammation. J Allergy Clin Immunol (2014) 134(6):1301–9.e11. 10.1016/j.jaci.2014.07.059 25262465

[29] Zeeuwen PL Ederveen TH van der Krieken DA Niehues H Boekhorst J Kezic S Gram-positive anaerobe cocci are underrepresented in the microbiome of filaggrin-deficient human skin. J Allergy Clin Immunol (2017) 139(4):1368–71. 10.1016/j.jaci.2016.09.017 27725187

[30] Arweiler NB Netuschil L. The oral microbiota. Adv Exp Med Biol (2016) 902:45–60. 10.1007/978-3-319-31248-4_4 27161350

[31] Costalonga M Herzberg MC. The oral microbiome and the immunobiology of periodontal disease and caries. Immunol Lett (2014) 162(2 Pt A):22–38. 10.1016/j.imlet.2014.08.017 25447398 PMC4346134

[32] Listgarten MA Microorganisms and dental implants. J Periodontol (1999) 70(2):220–2. 10.1902/jop.1999.70.2.220 10102562

[33] Socransky SS Haffajee AD Periodontal microbial ecology. Periodontol 2000 (2005) 38:135–87. 10.1111/j.1600-0757.2005.00107.x 15853940

[34] Hall MW Singh N Ng KF Lam DK Goldberg MB Tenenbaum HC Inter-personal diversity and temporal dynamics of dental, tongue, and salivary microbiota in the healthy oral cavity. NPJ Biofilms Microbiomes (2017) 3:2. 10.1038/s41522-016-0011-0 28649403 PMC5445578

[35] Takeshita T Kageyama S Furuta M Tsuboi H Takeuchi K Shibata Y Bacterial diversity in saliva and oral health-related conditions: the Hisayama Study. Sci Rep (2016) 6:22164. 10.1038/srep22164 26907866 PMC4764907

[36] Dewhirst FE Chen T Izard J Paster BJ Tanner AC Yu WH The human oral microbiome. J Bacteriol (2010) 192(19):5002–17. 10.1128/JB.00542-10 20656903 PMC2944498

[37] Wescombe PA Heng NC Burton JP Chilcott CN Tagg JR. Streptococcal bacteriocins and the case for *Streptococcus salivarius* as model oral probiotics. Future Microbiol (2009) 4(7):819–35. 10.2217/fmb.09.61 19722837

[38] Burton JP Wescombe PA Moore CJ Chilcott CN Tagg JR. Safety assessment of the oral cavity probiotic *Streptococcus salivarius* K12. Appl Environ Microbiol (2006) 72(4):3050–3. 10.1128/AEM.72.4.3050-3053.2006 16598017 PMC1449041

[39] Burton JP Chilcott CN Moore CJ Speiser G Tagg JR A preliminary study of the effect of probiotic *Streptococcus salivarius* K12 on oral malodour parameters. J Appl Microbiol (2006) 100(4):754–64. 10.1111/j.1365-2672.2006.02837.x 16553730

[40] Griffen AL Beall CJ Campbell JH Firestone ND Kumar PS Yang ZK Distinct and complex bacterial profiles in human periodontitis and health revealed by 16S pyrosequencing. ISME J (2012) 6(6):1176–85. 10.1038/ismej.2011.191 22170420 PMC3358035

[41] Vartoukian SR Palmer RM Wade WG Diversity and morphology of members of the phylum “synergistetes” in periodontal health and disease. Appl Environ Microbiol (2009) 75(11):3777–86. 10.1128/AEM.02763-08 19346352 PMC2687275

[42] Kumar PS Griffen AL Barton JA Paster BJ Moeschberger ML Leys EJ. New bacterial species associated with chronic periodontitis. J Dent Res (2003) 82(5):338–44. 10.1177/154405910308200503 12709498

[43] Ross KF Herzberg MC. Calprotectin expression by gingival epithelial cells. Infect Immun (2001) 69(5):3248–54. 10.1128/IAI.69.5.3248-3254.2001 11292747 PMC98283

[44] Burns E Bachrach G Shapira L Nussbaum G. Cutting edge: TLR2 is required for the innate response to *Porphyromonas gingivalis*: activation leads to bacterial persistence and TLR2 deficiency attenuates induced alveolar bone resorption. J Immunol (2006) 177(12):8296–300. 10.4049/jimmunol.177.12.8296 17142724

[45] Jiao Y Darzi Y Tawaratsumida K Marchesan JT Hasegawa M Moon H Induction of bone loss by pathobiont-mediated NOD1 signaling in the oral cavity. Cell Host Microbe (2013) 13(5):595–601. 10.1016/j.chom.2013.04.005 23684310 PMC3721316

[46] Hiroshima Y Sakamoto E Yoshida K Abe K Naruishi K Yamamoto T Advanced glycation end-products and *Porphyromonas gingivalis* lipopolysaccharide increase calprotectin expression in human gingival epithelial cells. J Cell Biochem (2018) 119(2):1591–603. 10.1002/jcb.26319 28771806

[47] Dutzan N Abusleme L Bridgeman H Greenwell-Wild T Zangerle-Murray T Fife ME On-going mechanical damage from mastication drives homeostatic Th17 cell responses at the oral barrier. Immunity (2017) 46(1):133–47. 10.1016/j.immuni.2016.12.010 28087239 PMC5263257

[48] Lamont RJ Hajishengallis G Polymicrobial synergy and dysbiosis in inflammatory disease. Trends Mol Med (2015) 21(3):172–83. 10.1016/j.molmed.2014.11.004 25498392 PMC4352384

[49] Glowczyk I Wong A Potempa B Babyak O Lech M Lamont RJ Inactive gingipains from *P. gingivalis* selectively skews T cells toward a Th17 phenotype in an IL-6 dependent manner. Front Cell Infect Microbiol (2017) 7:140. 10.3389/fcimb.2017.00140 28497028 PMC5406403

[50] Moutsopoulos NM Konkel J Sarmadi M Eskan MA Wild T Dutzan N Defective neutrophil recruitment in leukocyte adhesion deficiency type I disease causes local IL-17-driven inflammatory bone loss. Sci Transl Med (2014) 6(229):229ra40. 10.1126/scitranslmed.3007696 24670684 PMC4090608

[51] Mysak J Podzimek S Sommerova P Lyuya-Mi Y Bartova J Janatova T *Porphyromonas gingivalis*: major periodontopathic pathogen overview. J Immunol Res (2014) 2014:476068. 10.1155/2014/476068 24741603 PMC3984870

[52] Hajishengallis G Liang S Payne MA Hashim A Jotwani R Eskan MA Low-abundance biofilm species orchestrates inflammatory periodontal disease through the commensal microbiota and complement. Cell Host Microbe (2011) 10(5):497–506. 10.1016/j.chom.2011.10.006 22036469 PMC3221781

[53] Maekawa T Krauss JL Abe T Jotwani R Triantafilou M Triantafilou K *Porphyromonas gingivalis* manipulates complement and TLR signaling to uncouple bacterial clearance from inflammation and promote dysbiosis. Cell Host Microbe (2014) 15(6):768–78. 10.1016/j.chom.2014.05.012 24922578 PMC4071223

[54] Uriarte SM Edmisson JS Jimenez-Flores E. Human neutrophils and oral microbiota: a constant tug-of-war between a harmonious and a discordant coexistence. Immunol Rev (2016) 273(1):282–98. 10.1111/imr.12451 27558341 PMC5353849

[55] Tada H Matsuyama T Nishioka T Hagiwara M Kiyoura Y Shimauchi H *Porphyromonas gingivalis* gingipain-dependently enhances IL-33 production in human gingival epithelial cells. PLoS One (2016) 11(4):e0152794. 10.1371/journal.pone.0152794 27058037 PMC4825981

[56] Tada H Shimizu T Matsushita K Takada H. *Porphyromonas gingivalis*-induced IL-33 down-regulates hCAP-18/LL-37 production in human gingival epithelial cells. Biomed Res (2017) 38(3):167–73. 10.2220/biomedres.38.167 28637951

[57] Laperine O Cloitre A Caillon J Huck O Bugueno IM Pilet P Interleukin-33 and RANK-l interplay in the alveolar bone loss associated to periodontitis. PLoS One (2016) 11(12):e0168080. 10.1371/journal.pone.0168080 27992569 PMC5167367

[58] Wang M Krauss JL Domon H Hosur KB Liang S Magotti P Microbial hijacking of complement-toll-like receptor crosstalk. Sci Signal (2010) 3(109):ra11. 10.1126/scisignal.2000697 20159852 PMC2824906

[59] Liang S Krauss JL Domon H McIntosh ML Hosur KB Qu H The C5a receptor impairs IL-12-dependent clearance of *Porphyromonas gingivalis* and is required for induction of periodontal bone loss. J Immunol (2011) 186(2):869–77. 10.4049/jimmunol.1003252 21149611 PMC3075594

[60] Hajishengallis G Shakhatreh MA Wang M Liang S. Complement receptor 3 blockade promotes IL-12-mediated clearance of *Porphyromonas gingivalis* and negates its virulence in vivo. J Immunol (2007) 179(4):2359–67. 10.4049/jimmunol.179.4.2359 17675497

[61] Hajishengallis G. The inflammophilic character of the periodontitis-associated microbiota. Mol Oral Microbiol (2014) 29(6):248–57. 10.1111/omi.12065 24976068 PMC4232466

[62] Ebersole JL Dawson D III Emecen-Huja P Nagarajan R Howard K Grady ME The periodontal war: microbes and immunity. Periodontol 2000 (2017) 75(1):52–115. 10.1111/prd.12222 28758303

[63] Huang CB Alimova Y Ebersole JL. Macrophage polarization in response to oral commensals and pathogens. Pathog Dis (2016) 74(3):1–10. 10.1093/femspd/ftw011 26884502 PMC5975235

[64] Jotwani R Pulendran B Agrawal S Cutler CW. Human dendritic cells respond to *Porphyromonas gingivalis* LPS by promoting a Th2 effector response in vitro. Eur J Immunol (2003) 33(11):2980–6. 10.1002/eji.200324392 14579266

[65] Gonzales JR Groger S Boedeker RH Meyle J. Expression and secretion levels of Th1 and Th2 cytokines in patients with aggressive periodontitis. Clin Oral Investig (2012) 16(5):1463–73. 10.1007/s00784-011-0634-8 22065246

[66] Gonzales JR Groeger S Johansson A Meyle J T helper cells from aggressive periodontitis patients produce higher levels of interleukin-1 beta and interleukin-6 in interaction with *Porphyromonas gingivalis*. Clin Oral Investig (2014) 18(7):1835–43. 10.1007/s00784-013-1162-5 24352581

[67] Vallor AC Antonio MA Hawes SE Hillier SL Factors associated with acquisition of, or persistent colonization by, vaginal lactobacilli: role of hydrogen peroxide production. J Infect Dis (2001) 184(11):1431–6. 10.1086/324445 11709785

[68] Hawes SE Hillier SL Benedetti J Stevens CE Koutsky LA Wolner-Hanssen P Hydrogen peroxide-producing lactobacilli and acquisition of vaginal infections. J Infect Dis (1996) 174(5):1058–63. 10.1093/infdis/174.5.1058 8896509

[69] Gajer P Brotman RM Bai G Sakamoto J Schutte UM Zhong X Temporal dynamics of the human vaginal microbiota. Sci Transl Med (2012) 4(132):132ra52 10.1126/scitranslmed.3003605 PMC3722878 22553250

[70] Ravel J Gajer P Abdo Z Schneider GM Koenig SS McCulle SL Vaginal microbiome of reproductive-age women. Proc Natl Acad Sci U S A (2011) 108(Suppl 1):4680–7. 10.1073/pnas.1002611107 20534435 PMC3063603

[71] Doerflinger SY Throop AL Herbst-Kralovetz MM. Bacteria in the vaginal microbiome alter the innate immune response and barrier properties of the human vaginal epithelia in a species-specific manner. J Infect Dis (2014) 209(12):1989–99. 10.1093/infdis/jiu004 24403560

[72] Verstraelen H Verhelst R Nuytinck L Roelens K De Meester E De Vos D Gene polymorphisms of toll-like and related recognition receptors in relation to the vaginal carriage of *Gardnerella vaginalis* and *Atopobium vaginae*. J Reprod Immunol (2009) 79(2):163–73. 10.1016/j.jri.2008.10.006 19200604

[73] Jones NM Holzman C Friderici KH Jernigan K Chung H Wirth J Interplay of cytokine polymorphisms and bacterial vaginosis in the etiology of preterm delivery. J Reprod Immunol (2010) 87(1–2):82–9. 10.1016/j.jri.2010.06.158 20965572 PMC3005194

[74] Sanu O Lamont RF. Periodontal disease and bacterial vaginosis as genetic and environmental markers for the risk of spontaneous preterm labor and preterm birth. J Matern Fetal Neonatal Med (2011) 24(12):1476–85. 10.3109/14767058.2010.545930 21261445

[75] Horne AW Stock SJ King AE Innate immunity and disorders of the female reproductive tract. Reproduction (2008) 135(6):739–49. 10.1530/REP-07-0564 18502890

[76] Usluogullari B Gumus I Gunduz E Kaygusuz I Simavli S Acar M The role of human dectin-1 Y238X gene polymorphism in recurrent vulvovaginal candidiasis infections. Mol Biol Rep (2014) 41(10):6763–8. 10.1007/s11033-014-3562-2 25008994

[77] Rose WA II McGowin CL Spagnuolo RA Eaves-Pyles TD Popov VL Pyles RB. Commensal bacteria modulate innate immune responses of vaginal epithelial cell multilayer cultures. PLoS One (2012) 7(3):e32728. 10.1371/journal.pone.0032728 22412914 PMC3296736

[78] Anahtar MN Byrne EH Doherty KE Bowman BA Yamamoto HS Soumillon M Cervicovaginal bacteria are a major modulator of host inflammatory responses in the female genital tract. Immunity (2015) 42(5):965–76. 10.1016/j.immuni.2015.04.019 25992865 PMC4461369

[79] Shin H Iwasaki A. Tissue-resident memory T cells. Immunol Rev (2013) 255(1):165–81. 10.1111/imr.12087 23947354 PMC3748618

[80] Smith SB Ravel J. The vaginal microbiota, host defence and reproductive physiology. J Physiol (2017) 595(2):451–63. 10.1113/JP271694 27373840 PMC5233653

[81] Turner MW. The role of mannose-binding lectin in health and disease. Mol Immunol (2003) 40(7):423–9. 10.1016/S0161-5890(03)00155-X 14568388

[82] Babula O Lazdane G Kroica J Ledger WJ Witkin SS. Relation between recurrent vulvovaginal candidiasis, vaginal concentrations of mannose-binding lectin, and a mannose-binding lectin gene polymorphism in Latvian women. Clin Infect Dis (2003) 37(5):733–7. 10.1086/377234 12942410

[83] Khan FF Carpenter D Mitchell L Mansouri O Black HA Tyson J Accurate measurement of gene copy number for human alpha-defensin DEFA1A3. BMC Genomics (2013) 14:719. 10.1186/1471-2164-14-719 24138543 PMC4046698

[84] Valore EV Wiley DJ Ganz T. Reversible deficiency of antimicrobial polypeptides in bacterial vaginosis. Infect Immun (2006) 74(10):5693–702. 10.1128/IAI.00524-06 16988245 PMC1594936

[85] Wang YY Kannan A Nunn KL Murphy MA Subramani DB Moench T IgG in cervicovaginal mucus traps HSV and prevents vaginal herpes infections. Mucosal Immunol (2014) 7(5):1036–44. 10.1038/mi.2013.120 24496316 PMC4122653

[86] Breshears LM Edwards VL Ravel J Peterson ML. *Lactobacillus crispatus* inhibits growth of *Gardnerella vaginalis* and *Neisseria gonorrhoeae* on a porcine vaginal mucosa model. BMC Microbiol (2015) 15:276. 10.1186/s12866-015-0608-0 26652855 PMC4675025

[87] Mastromarino P Di Pietro M Schiavoni G Nardis C Gentile M Sessa R Effects of vaginal lactobacilli in *Chlamydia trachomatis* infection. Int J Med Microbiol (2014) 304(5–6):654–61. 10.1016/j.ijmm.2014.04.006 24875405

[88] Buve A Jespers V Crucitti T Fichorova RN The vaginal microbiota and susceptibility to HIV. AIDS (2014) 28(16):2333–44. 10.1097/QAD.0000000000000432 25389548

[89] Nahui Palomino RA Zicari S Vanpouille C Vitali B Margolis L. Vaginal *Lactobacillus* inhibits HIV-1 replication in human tissues ex vivo. Front Microbiol (2017) 8:906. 10.3389/fmicb.2017.00906 28579980 PMC5437121

[90] Wang S Wang Q Yang E Yan L Li T Zhuang H. Antimicrobial compounds produced by vaginal *Lactobacillus crispatus* are able to strongly inhibit *Candida albicans* growth, hyphal formation and regulate virulence-related gene expressions. Front Microbiol (2017) 8:564. 10.3389/fmicb.2017.00564 28421058 PMC5378977

[91] Hearps AC Tyssen D Srbinovski D Bayigga L Diaz DJD Aldunate M Vaginal lactic acid elicits an anti-inflammatory response from human cervicovaginal epithelial cells and inhibits production of pro-inflammatory mediators associated with HIV acquisition. Mucosal Immunol (2017) 10(6):1480–90. 10.1038/mi.2017.27 28401934

[92] Donders GG Vereecken A Bosmans E Dekeersmaecker A Salembier G Spitz B. Definition of a type of abnormal vaginal flora that is distinct from bacterial vaginosis: aerobic vaginitis. BJOG (2002) 109(1):34–43. 10.1111/j.1471-0528.2002.00432.x 11845812

[93] Donders G Bellen G Rezeberga D. Aerobic vaginitis in pregnancy. BJOG (2011) 118(10):1163–70. 10.1111/j.1471-0528.2011.03020.x 21668769

[94] Edwards L. Dermatologic causes of vaginitis: a clinical review. Dermatol Clin (2010) 28(4):727–35. 10.1016/j.det.2010.07.004 20883916

[95] Amsel R Totten PA Spiegel CA Chen KC Eschenbach D Holmes KK Nonspecific vaginitis. Diagnostic criteria and microbial and epidemiologic associations. Am J Med (1983) 74(1):14–22. 10.1016/0002-9343(83)91112-9 6600371

[96] Nugent RP Krohn MA Hillier SL. Reliability of diagnosing bacterial vaginosis is improved by a standardized method of Gram stain interpretation. J Clin Microbiol (1991) 29(2):297–301. 1706728 10.1128/jcm.29.2.297-301.1991 PMC269757

[97] Mitchell C Marrazzo J. Bacterial vaginosis and the cervicovaginal immune response. Am J Reprod Immunol (2014) 71(6):555–63. 10.1111/aji.12264 24832618 PMC4128638

[98] Lamont RF Sobel JD Akins RA Hassan SS Chaiworapongsa T Kusanovic JP The vaginal microbiome: new information about genital tract flora using molecular based techniques. BJOG (2011) 118(5):533–49. 10.1111/j.1471-0528.2010.02840.x 21251190 PMC3055920

[99] Mendling W. Vaginal microbiota. Adv Exp Med Biol (2016) 902:83–93. 10.1007/978-3-319-31248-4_6 27161352

[100] Consolandi C Turroni S Emmi G Severgnini M Fiori J Peano C Behcet’s syndrome patients exhibit specific microbiome signature. Autoimmun Rev (2015) 14(4):269–76. 10.1016/j.autrev.2014.11.009 25435420

[101] Mirmonsef P Zariffard MR Gilbert D Makinde H Landay AL Spear GT. Short-chain fatty acids induce pro-inflammatory cytokine production alone and in combination with toll-like receptor ligands. Am J Reprod Immunol (2012) 67(5):391–400. 10.1111/j.1600-0897.2011.01089.x 22059850 PMC3288536

[102] Aldunate M Srbinovski D Hearps AC Latham CF Ramsland PA Gugasyan R Antimicrobial and immune modulatory effects of lactic acid and short chain fatty acids produced by vaginal microbiota associated with eubiosis and bacterial vaginosis. Front Physiol (2015) 6:164. 10.3389/fphys.2015.00164 26082720 PMC4451362

[103] Eade CR Diaz C Wood MP Anastos K Patterson BK Gupta P Identification and characterization of bacterial vaginosis-associated pathogens using a comprehensive cervical-vaginal epithelial coculture assay. PLoS One (2012) 7(11):e50106. 10.1371/journal.pone.0050106 23166828 PMC3499514

[104] Thurman AR Kimble T Herold B Mesquita PM Fichorova RN Dawood HY Bacterial vaginosis and subclinical markers of genital tract inflammation and mucosal immunity. AIDS Res Hum Retroviruses (2015) 31(11):1139–52. 10.1089/aid.2015.0006 26204200 PMC4651020

[105] Fichorova RN Lai JJ Schwartz JL Weiner DH Mauck CK Callahan MM. Baseline variation and associations between subject characteristics and five cytokine biomarkers of vaginal safety among healthy non-pregnant women in microbicide trials. Cytokine (2011) 55(1):134–40. 10.1016/j.cyto.2011.03.016 21530305

[106] Rebbapragada A Howe K Wachihi C Pettengell C Sunderji S Huibner S Bacterial vaginosis in HIV-infected women induces reversible alterations in the cervical immune environment. J Acquir Immune Defic Syndr (2008) 49(5):520–2. 10.1097/QAI.0b013e318189a7ca 18989228

[107] Alcaide ML Strbo N Romero L Jones DL Rodriguez VJ Arheart K Bacterial vaginosis is associated with loss of gamma delta T cells in the female reproductive tract in women in the Miami Women Interagency HIV Study (WIHS): a cross sectional study. PLoS One (2016) 11(4):e0153045. 10.1371/journal.pone.0153045 27078021 PMC4831836

[108] Kyongo JK Jespers V Goovaerts O Michiels J Menten J Fichorova RN Searching for lower female genital tract soluble and cellular biomarkers: defining levels and predictors in a cohort of healthy Caucasian women. PLoS One (2012) 7(8):e43951. 10.1371/journal.pone.0043951 22952818 PMC3432048

[109] Oh JE Kim BC Chang DH Kwon M Lee SY Kang D Dysbiosis-induced IL-33 contributes to impaired antiviral immunity in the genital mucosa. Proc Natl Acad Sci U S A (2016) 113(6):E762–71. 10.1073/pnas.1518589113 26811463 PMC4760794

[110] Oh JE Oh DS Jung HE Lee HK. A mechanism for the induction of type 2 immune responses by a protease allergen in the genital tract. Proc Natl Acad Sci U S A (2017) 114(7):E1188–95. 10.1073/pnas.1612997114 28137851 PMC5320955

[111] Atarashi K Suda W Luo C Kawaguchi T Motoo I Narushima S Ectopic colonization of oral bacteria in the intestine drives TH1 cell induction and inflammation. Science (2017) 358(6361):359–65. 10.1126/science.aan4526 29051379 PMC5682622

[112] Coit P Mumcu G Ture-Ozdemir F Unal AU Alpar U Bostanci N Sequencing of 16S rRNA reveals a distinct salivary microbiome signature in Behcet’s disease. Clin Immunol (2016) 169:28–35. 10.1016/j.clim.2016.06.002 27283393

[113] Mueller SN Gebhardt T Carbone FR Heath WR. Memory T cell subsets, migration patterns, and tissue residence. Annu Rev Immunol (2013) 31:137–61. 10.1146/annurev-immunol-032712-095954 23215646

[114] Park JY Chung H Choi Y Park JH. Phenotype and tissue residency of lymphocytes in the murine oral mucosa. Front Immunol (2017) 8:250. 10.3389/fimmu.2017.00250 28337201 PMC5340784

[115] Posavad CM Zhao L Dong L Jin L Stevens CE Magaret AS Enrichment of herpes simplex virus type 2 (HSV-2) reactive mucosal T cells in the human female genital tract. Mucosal Immunol (2017) 10(5):1259–69. 10.1038/mi.2016.118 28051084 PMC5496807

[116] Park CO Kupper TS. The emerging role of resident memory T cells in protective immunity and inflammatory disease. Nat Med (2015) 21(7):688–97. 10.1038/nm.3883 26121195 PMC4640452

